# Exploring the pharmacokinetic mechanisms that affect bictegravir exposure during pregnancy

**DOI:** 10.1093/jac/dkag159

**Published:** 2026-05-06

**Authors:** L van der Wekken-Pas, C Hidalgo-Tenorio, J K Rockstroh, K van Bremen, O Richel, J Molto, J S Lambert, C de Kanter, D Konopnicki, D Burger, A Colbers

**Affiliations:** Department of Pharmacy, Pharmacology and Toxicology, Radboudumc, Nijmegen, The Netherlands; Infectious Diseases Unit, Hospital Universitario Virgen de las Nieves, Instituto de Investigación Biosanitario de Granada (IBS-Grana), Granada, Spain; Department of Internal Medicine, Universitätsklinikum Bonn, Bonn, Germany; Department of Internal Medicine, Universitätsklinikum Bonn, Bonn, Germany; Department of Internal Medicine, Radboudumc, Nijmegen, The Netherlands; Department of Infectious Diseases, Hospital Universitari Germans Trias I Pujol and Fundació Lluita Contra les Infeccions, Badalona, Spain and CIBERINFEC, Instituto de Salud Carlos III, Madrid, Spain; Department of Infectious Diseases, Saint James Hospital, Dublin, Ireland; Department of Pharmacy, Curaçao Medical Center, Willemstad, Curaçao; Infectious Diseases Department, Saint-Pierre University Hospital, Brussels, Belgium; Department of Pharmacy, Pharmacology and Toxicology, Radboudumc, Nijmegen, The Netherlands; Department of Pharmacy, Pharmacology and Toxicology, Radboudumc, Nijmegen, The Netherlands

## Abstract

**Introduction:**

Pregnancy is associated with physiological changes, resulting in altered pharmacokinetics, which may impact antiretroviral drug exposure. To assure health and prevent vertical transmission of HIV, it is imperative to reach adequate drug exposure. This study aims to determine the impact of pregnancy on bictegravir pharmacokinetics, examine mechanisms responsible for lower exposure, report on placental transfer, safety, and efficacy.

**Materials and methods:**

This open-label pharmacokinetic study, included women living with HIV who used bictegravir, emtricitabine with tenofovir alafenamide. Plasma samples were obtained in third trimester, as well as 4–6 weeks postpartum. If feasible, cord blood and maternal plasma at delivery were obtained.

**Results:**

Sixteen participants were included. Geometric mean area under the curve for total bictegravir was 48.8 mg*h/L (coefficient of variation (CV) 25.9%) in third trimester and 99.2 mg*L/h (CV 32.9%) postpartum, with a geometric mean ratio (90% CI) of 0.49 (0.44–0.55); 24 h post-dosing, median (IQR) unbound fractions were 0.15 (0.13–0.18)% and 0.11 (0.10–0.13) during pregnancy and postpartum, respectively. Median (IQR) ratio between pregnancy and postpartum was 173.7% (127.8–305.7) for glucuronidation-metabolite (M15) and 200.7% (150.0–224.9) for sulfation-metabolite (M20). Low-level viraemia was noted in several participants, but no vertical transmission occurred. Cord blood maternal plasma ratio (IQR) was 1.3 (1.0–1.4). No congenital anomalies were reported.

**Conclusion:**

Although bictegravir exposure is decreased during pregnancy, mainly due to altered protein binding and increased glucuronidation and sulfation, trough levels remained above the PA-IC95. No vertical transmission occurred and no congenital anomalities were observed. Bictegravir was shown to have profound placental transfer.

## Introduction

Worldwide, 1.3 million women living with HIV become pregnant each year.^1^ With the use of combined antiretroviral therapy (cART) during pregnancy and thereafter, rates of vertical transmission have dropped significantly.^2^ Daily intake of cART is vital to maintain maternal health and prevent vertical transmission. However, to be effective, adequate exposure is warranted. Pregnancy-related symptoms (nausea, fatigue, psychological symptoms) may hamper daily intake. And even if adequate intake is ensured, pharmacokinetic changes might influence the exposure thereafter.

Bictegravir (BIC) might be an appealing drug to be used in pregnancy, as it is available as a single tablet regimen (co-formulated with emtricitabine (FTC) and tenofovir alafenamide fumarate (TAF) (B/F/TAF; Biktarvy®), and it has a favourable side-effect profile. Bictegravir is an integrase strand transfer inhibitor and has a high genetic barrier, low potential for drug interactions, and is compatible with kidney dysfunction and hepatitis B co-infection. For this reason, this drug combination is now appointed as a preferred regimen in current guidelines.^3,4^

Earlier reports^5–7^ have shown lower exposure to bictegravir in the second and third trimester. Whereas the exact mechanism leading to this lower exposure to bictegravir has not yet been completely revealed, further research is warranted. Uridine diphosphate glucuronosyltransferase (UGT) 1A1 and cytochrome P450 (CYP) 3A4 are enzymes involved in the metabolization of bictegravir into inactive metabolites (M15 and M20, respectively), and they are known to be induced during pregnancy, potentially leading to lower drug concentrations. Additionally, changes in protein binding and changes in distribution volume caused by increased blood volume and total body water during pregnancy might contribute to changed exposure to bictegravir.^8–10^

Besides efficacy, safety is an important aspect to assess when considering a drug to be appropriate in pregnancy. Ex vivo placenta perfusion studies^11^ showed low placental transfer, which is contradicted by a case report by Le et al^12^ and other studies.^6,7^ Additional data is necessary to establish to what extent bictegravir crosses the placenta.

Considering all this, this study aims to describe bictegravir total and unbound concentrations and its metabolic ratios for the main metabolites in pregnancy compared with a non-pregnant state in women living with HIV, as well as placental transfer.

## Materials and methods

### Study design

This is a non-randomized, multicentre, open-label pharmacokinetic study to determine the exposure to bictegravir in pregnancy. Approval was obtained from the appropriate medical-ethical committees of each participating centre and, if necessary, also from the competent authorities. Prior to study procedures, informed consent was obtained from the participants. The trial is registered at ClinicalTrials.gov (identifier NCT00825929) and was designed and coordinated by the PANNA (Pharmacokinetics of Newly developed Antiretroviral Agents in Pregnant Women living with HIV) network study group. PANNA aimed to prospectively collect pharmacokinetic data on newly developed antiretroviral drugs in pregnant women living with HIV (pannastudy.com). Participants were recruited from 21 hospitals in 7 countries in Europe, the United Kingdom, and Curacao between March 2020 and December 2024. This report concerns bictegravir, co-formulated with emtricitabine and tenofovir alafenamide fumarate.

### Participants

Participants were eligible if they were pregnant, had an HIV-1 infection, and were at least 18 years of age and used BIC/TAF/FTC for a minimum of 2 weeks prior to the PK assessment in the third trimester to ensure steady state conditions for pharmacokinetic sampling. Participants were excluded in case of medical history or current condition that might interfere with absorption, distribution, metabolism, and elimination of bictegravir (such as renal or hepatic failure) and in case of grade III/IV anaemia (i.e. Hb <4.6 mmol/L or <7.4 g/dL). Intake of grapefruit and Seville oranges containing foods was prohibited for 7 days before the pharmacokinetic assessment.

### Procedures

The primary endpoint of the study was the comparison of PK parameters in the third trimester of pregnancy compared with a non-pregnant state. To this end, participants served as their own control by repeating the intensive pharmacokinetic assessment 4–6 weeks postpartum. The PK assessment in the third trimester took place preferably at 33 weeks of gestational age.

After a standardized breakfast (650 kcal and 30 g fat), samples were collected at the following time points: pre-dose and 0.5, 1, 2, 3, 4, 6, 8, 12, and 24 h after drug intake.

Total and unbound concentrations were measured 2- and 24-h post-dosing, to ensure capture of the highest and lowest concentrations. Bictegravir concentrations were determined using liquid chromatography with tandem mass spectrometry (LC-MS/MS) with a lower and upper limit of detection of 0.02 and 20 mg/L, respectively.^13^ Glucuronidation- and sulfation metabolites of bictegravir—M15 and M20, respectively—were also assessed using LC-MS/MS. Lower and upper limit of detection for M15 and M20 were 0.002 and 2.0 mg/L, respectively. These assays have been developed in accordance with guidance from the European Medicines Agency (EMA guideline bioanalytical methods).^14^ The metabolic ratio was calculated for each metabolite by dividing the area under the curve (AUC) of the metabolite of interest by the AUC of total bictegravir, both in pregnancy and postpartum. Subsequently, the ratio in pregnancy was divided by the ratio postpartum for both M15 and M20 to calculate the pregnancy:postpartum ratio for each metabolite. The assay for quantification of total bictegravir concentrations is externally validated by the International Interlaboratory Program for the Quality Control of Therapeutic Drug Monitoring in HIV Treatment.^15,16^

Other parameters obtained in this study were infant birth weight, gestational age at birth, congenital abnormalities, and HIV infection status (HIV RNA 1 week after birth). Each visit, participants were asked if they experienced side effects and the reported (serious) adverse events were graded using the DAIDS table.^17^ If circumstances allowed, paired cord blood and maternal blood were obtained after delivery to determine placental transfer.

### Statistical analysis

PK parameters were computed with non-compartmental analysis using Winnonlin (Phoenix 64 version 8.4, Certara). The following parameters were calculated: AUC_0-24_, *C*_trough_, *C*_max_, and *T*_1/2_ as geometric means (GM) with percentage of coefficient of variation (CV%) and *T*_max_ as median (IQR). Unbound concentrations and fractions unbound at 2- and 24-h post-dosing are reported as geometric mean (CV%) and median (IQR), respectively. The metabolic ratio for M15 and M20 was determined based on the AUC_last_ of total bictegravir and its metabolite in μmol*h/L. Geometric mean ratio (GMR) with 90% confidence intervals (90% CI) for bictegravir concentrations in pregnancy compared with postpartum was determined based on log-transformed pharmacokinetic parameters using a linear mixed model (pregnancy set as fixed effect and participant as random effect). Descriptive statistics were performed for demographic, efficacy, and safety data with the use of R/Rstudio (version 2025.09.0 + 387).

## Results

### Participants

Sixteen women were included from 10 sites in 6 different countries (the Netherlands, Belgium, Germany, Spain, Curaçao, and Ireland). The characteristics of the participants and their infants are summarized in Table 1. For the calculation of the AUC_0-24h_, half-life and *C*_trough_14 concentration–time curves were eligible, whereas curves from all 16 participants were used to determine *C*_max_ and *T*_max._

**Table 1.** Characteristics of the participants

	Median (IQR) or number (%)
Age years	32.5 (26.7–34.8)
Weight, kg	
At inclusion	68.0 (60.1–84.9)
Post partum	63.3 (56.5–79.3)
Ethnicity	
White	7 (43.8)
Black	7 (43.8)
Asian	0 (0)
Other	2 (12.5)
CD4 count at inclusion, cells/*μ*	565 (349–775.5)
Time since start or switch to B/F/TAF, weeks	89.7 (57.1–114.1)
Serum creatinine, µmol/L	
During pregnancy	48.8 (45.3–57.5)
Post partum	70.5 (53.1–81.8)
Serum albumin, g/L	
During pregnancy	32.0 (28.0–35.4)
Post partum	38.0 (37.0–40.3)
Serum alpha-1-acid-glycoprotein, g/L	
During pregnancy	4.5 (3.0–5.8) (*n* = 5)
Post partum	6.1 (5.7–12) (*n* = 4)
Gestational age at delivery, weeks	40.4 (39.8–41)
Time post partum at time of sampling, weeks	5.2 (4.3–5.7)
Mode of delivery	
Caesarean section	6
Vaginal birth	10
Birth weight, gram	3186 (2694–3582)
Small for gestational age	0 (0)
Congenital anomalies	0 (0)
Vertical HIV transmissions	0 (0)

### Pharmacokinetic outcomes

**Figure 1.**
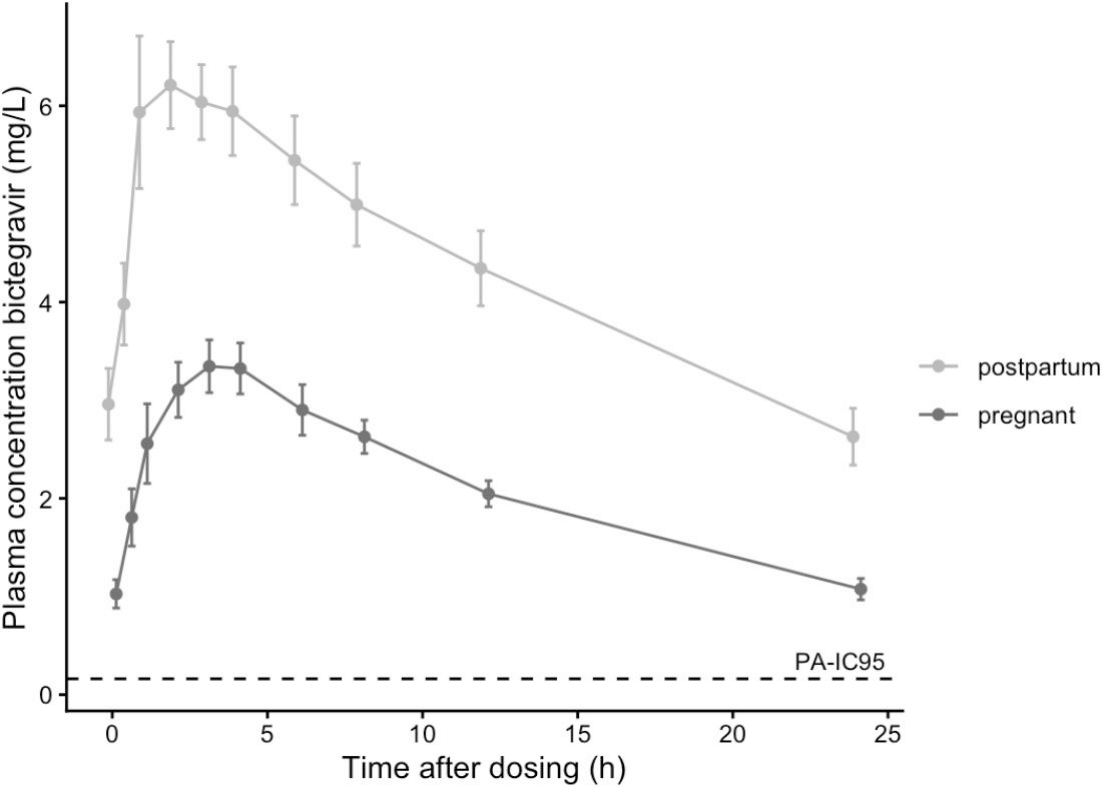
Mean (95% CI) total concentration of bictegravir (mg/L) in plasma during pregnancy and postpartum.

The concentrations of bictegravir over time in both pregnancy and postpartum are presented in Figure 1 and Table 2. Total exposure to bictegravir was 51% lower in pregnancy compared with postpartum. The AUC_0-24h_, *C*_max,_ and *C*_trough_ were lower in all participants during pregnancy compared with postpartum. Bictegravir elimination half-life was on average 27% shorter during pregnancy when compared with postpartum (see Figure S1A–D; available as Supplementary data at *JAC* Online).

**Table 2.** Geometric mean (CV%) pharmacokinetic parameters and or median (IQR) *T*_max_ in pregnancy and postpartum, and geometric mean ratios with 90% confidence intervals

	Third trimester	Postpartum	Geometric mean ratio (90% CI)
AUC_0-24_ (mg*h/L) (CV%)	48.8 (25.9)	99.2 (32.9)	0.49 (0.44–0.55)
*T*_max_ (h)	3 (1.5–3.0)	2 (1.3–3.8)	—
*C*_max_ (mg/L) (CV%)	4.0 (27.8)	6.9 (29.3)	0.57 (0.48–0.66)
*C*_trough_ (mg/L) (CV%)	1.0 (42.4)	2.42 (46.1)	0.41 (0.35–0.49)
*T*_1/2_ (h) (CV%)	12.2 (23.3)	16.7 (21.8)	0.73 (0.66–0.80)

**Figure 2.**
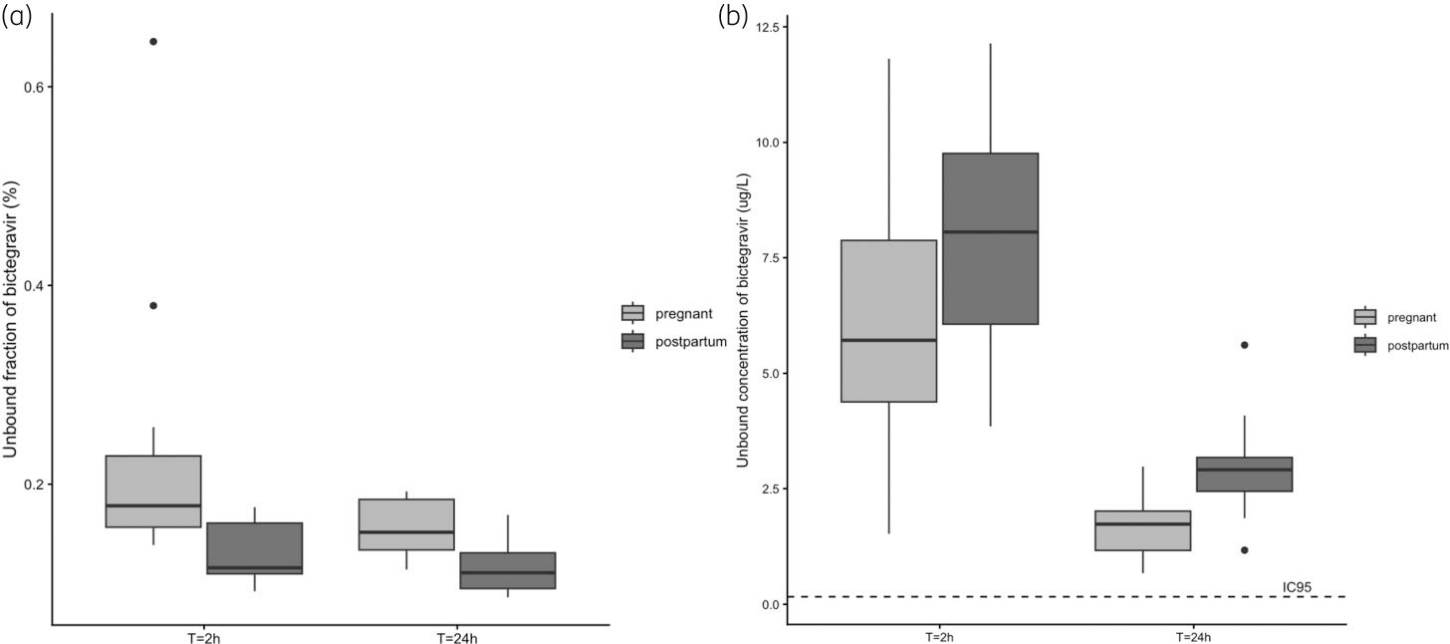
(a) Boxplot (median; IQR) of unbound fraction of bictegravir (%) in pregnancy and postpartum. (b) Boxplot (median; IQR) of the unbound concentration of bictegravir (µg/L) in pregnancy and postpartum.

In pregnancy, the unbound fractions were higher compared with postpartum. Median (IQR) unbound fractions were 0.18 (0.16–0.23)% 2 h post-dosing and 0.15 (0.13–0.18)% 24 h post-dosing during pregnancy, whereas they were 0.12 (0.11–0.16) and 0.11 (0.10–0.13) postpartum at these timepoints, respectively. The unbound concentrations were lower in pregnancy. The geometric mean (CV%) unbound concentration of bictegravir was 5.5 (54.8) μg/L 2 h after dosing and 1.5 (45.5) μg/L 24 h after dosing. At the postpartum visit, the unbound concentrations were 7.6 (35.8) μg/L 2 h after dosing and 2.7 (40.2) μg/L 24 h after dosing, respectively (Figure 2a and b).

The metabolic rate for both glucuronidation route (M15) and the sulfation route (M20) were increased during pregnancy. The geometric mean (CV%) AUC_last_ for the M15 and M20 metabolites of bictegravir of bictegravir and their metabolic rates both during pregnancy and postpartum are summarized in Table 3.

**Table 3.** Metabolic rates of metabolites of bictegravir (M15—result of glucuronidation and M20—result of sulfation) and their pregnancy: postpartum ratio

	Geometric mean AUC*last* (CV%) total BIC (μmol*h/L)	Geometric mean AUC*last* (CV%) M15 (μmol*h/L)	Median (IQR) metabolic ratio M15/BIC	Geometric mean AUC*last* (CV%) M20 (μmol*h/L)	Median (IQR) metabolic ratio M20/BIC
Pregnancy	108.6 (25.9)	4.2 (57.1)	0.03 (0.03–0.05)	19.6 (41.4)	0.18 (0.16–0.22)
Postpartum	220.8 (32.9)	3.8 (30.2)	0.02 (0.01–0.02)	19.9 (47.4)	0.09 (0.07–0.12)
Median (IQR) ratio pregnancy/postpartum	0.49 (43.8–55.2)	—	1.74 (1.28–3.06)	—	2.01 (1.50–2.25)

### Placental transfer

Cord blood and maternal plasma at delivery were obtained from four mother-infant pairs. The median (IQR) concentration in cord blood was 2.2 (1.8–2.6) mg/L, with a median (IQR) cord blood:maternal plasma ratio of 1.3 (1.0–1.4), indicating significant accumulation in neonatal plasma. Time between last drug intake and sampling ranged from 6 to 20 h.

### Clinical outcomes

HIV RNA was detected in several participants at different timepoints during the study period. Two participants had a detectable viral load in the third trimester (55 and 1001 copies/mL) and were resuppressed before delivery. Two other participants had 55 and 1480 copies/mL at the postpartum visit (summarized in Table 4). No information was available on drug adherence, but pre-dose concentration did not suggest non-adherence. As all participants resuppressed spontaneously, no resistance testing was performed. No vertical transmission occurred. Also, no congenital anomalies were reported. All six participants underwent a caesarean section because of obstetric reasons. Three serious adverse events were recorded, none of which were likely to be related to the treatment (non-urgent unplanned caesarean section, postpartum endometritis, complicated urinary tract infection)

**Table 4.** HIV RNA during the study period

	Undetectable (<50 copies/mL), number (%)	Detectable, number (%) (viral load in copies/mL)
3rd trimester visit	14/16 (87.5%)	2/16 (12.5%) (55, 1001)
Within 48 h of delivery	2/2 (100%)	0/16 (0%)
Postpartum visit	12/14 (85.7%)	2/14 (14.3%) (55, 1480)

## Discussion

Total bictegravir plasma concentrations were decreased during pregnancy when compared with postpartum. Nevertheless, all trough concentrations remained above the protein-adjusted inhibitory concentration (PA-IC_95_) of 0.162 mg/L for bictegravir. Also, bictegravir is highly protein-bound (>99*% in vitro*^18^), and even though unbound concentrations were also decreased during pregnancy, they remained far above this concentration, suggesting sufficient exposure. Although several participants exhibited detectable HIV RNA at various timepoints, none displayed detectable viral loads before delivery, and no cases of vertical transmission occurred. These findings are consistent with those from previous studies,^5–7^ further substantiating the pharmacokinetic behaviour of bictegravir in the context of pregnancy.

The decreased concentrations of bictegravir observed during pregnancy appear to be driven by multiple physiological changes. Notably, reductions in serum albumin and alpha-1-acid glycoprotein—both key binding proteins—were evident, likely as a consequence of the increased blood volume and total body water known to occur during pregnancy. This shift in protein binding was reflected by a higher unbound fraction of bictegravir. The unbound concentrations were also decreased during pregnancy, suggesting additional mechanisms are in play. Increased metabolism—illustrated by the increased rate of both glucuronidation by UGT1A1 into M15 and sulfation by CYP3A4 into M20, is probably responsible for this phenomenon. In male subjects, the M20 pathway is the main route for metabolization of bictegravir (20.1%), followed by the M15 pathway (8.6%).^19^ Both pathways are known to be induced in pregnancy, as was previously shown for drugs that are mainly metabolized by CYP3A4 (rilpivirine) and UGT1A1 (raltegravir), respectively. The decrease in exposure to bictegravir—a substrate for both enzymes—during pregnancy (AUC −51%) is bigger than seen in rilpivirine (AUC −45%)^20^ and raltegravir (AUC −29%),^21^ underlining the importance of both metabolic pathways and the interplay with shifts in protein binding.

Bictegravir is co-formulated with emtricitabine and tenofovir alafenamide—and even though exposure to these drugs is also impacted by pregnancy induced pharmacokinetic changes, it is not expected to influence efficacy. Tenofovir alafenamide has lower total concentrations in pregnancy,^22,23^ but the decrease in unbound drug is modest.^6^ Also, the trough concentration of the active tenofovir-diphosphate in peripheral blood mononuclear cells did not differ significantly in pregnant women when compared with postpartum state.^6^ Emtricitabine concentrations are also decreased in pregnancy,^24,25^ however, the magnitude of this decrease does not warrant dose adjustment in pregnancy.

Besides maternal exposure, neonatal exposure to bictegravir through placental transfer was also reported in this study. And even though extensive placental transfer was documented, no congenital anomalies were noted. Also, obstetric outcomes were positive, with no preterm labour or infants classified as small for gestational age. Despite low number of participants, as the study was primarily powered to assess pharmacokinetic outcomes, the aggregation of safety data from other cohorts and data from the antiretroviral pregnancy registry reinforces these reassuring findings.^6,7,26,27^

Findings of the current study, combined with previous reports,^5–7^ support the recent approval of a label change for Biktarvy® during pregnancy by the Food and Drug Administration and the European Medicines Agency. Also, this regimen is now included in current guidelines as a preferred option.^4,28^ Single tablet regimens help to lower pill burden and thereby increase adherence, which is crucial during pregnancy and thereafter. As other single tablet regimens are not always suitable during pregnancy, due to co-formulation with rilpivirine or cobicistat (suboptimal exposure during pregnancy) or doravirine (insufficient pregnancy data available), and others are not available in some regions (dolutegravir with lamivudine and tenofovir disoproxil fumarate), the availability of a bictegravir-containing single tablet regimen for this population is an important improvement.

This study’s findings regarding placental transfer prompt important questions for future research, particularly whether substantial neonatal exposure might suffice for perinatal prophylaxis. Existing studies^6,7,12,29^ have documented extended neonatal half-lives of several drugs due to immature metabolism, especially concerning UGT related metabolism. Secondly, it is suggested that the added value of postnatal prophylaxis (neonatal administration of zidovudine or nevirapine for 2–4 weeks) is negligible in low-risk cases.^30^ Further pharmacokinetic modelling and prospective clinical studies (for example NCT07055451) are therefore warranted to determine the value of perinatal prophylaxis provided *in utero* by maternal intake in scenarios with a low risk of HIV transfer.

Several limitations should be acknowledged. The sample size was relatively small, and the observational design limits the assessment of long-term clinical outcomes. Nonetheless, these findings contribute valuable evidence to a field that has historically lagged behind in data supporting antiretroviral use during pregnancy. Findings of the current study are published almost two years after the first report on bictegravir exposure during pregnancy,,^6^while Biktarvy® was first approved in non-pregnant individuals seven years ago. In this period, pregnant individuals living with HIV have been prescribed this drug with insufficient information to conduct an adequate benefit-risk assessment. Multiple networks^31^ and expert groups^32,33^ have made recommendations to accelerate the acquisition of efficacy, safety, and pharmacokinetic data in pregnant individuals. It is reassuring to notice that recent^34^ and ongoing trials (i.e. NCT04994509) with new compounds are currently conducted without stringent contraceptive measures and inclusion of pregnant and breastfeeding individuals. We hope this movement will grow and that other sponsors follow this pursuit to further achieve equity in drug research and treatment.

### Conclusion

Although exposure to bictegravir is decreased during pregnancy, due to altered protein binding and increased metabolism by CYP3A4 and UGT1A1, trough concentrations remained well above inhibitory concentrations and no signs of loss of efficacy are noted. Bictegravir does cross the placenta; however, no negative obstetric outcomes or congenital anomalies were noted in this study.

## Supplementary Material

dkag159_Supplementary_Data

## Data Availability

Data is available upon reasonable request.

## Transparency declarations

LW has received speaker grants from ViiV and a research grant from the Dutch Society of HIV professionals, all paid to the institution. AC has received research grants (paid to the institution) from ViiV Healthcare, Gilead Sciences, and Merck. DB has served on advisory boards for and received research grants from (all paid to the institution) ViiV Healthcare, Gilead Sciences and Merck. JM has received research funding, consultancy fees and lecture sponsorships from and have served on advisory boards for various laboratories (MSD, Gilead Sciences, ViiV Healthcare, Johnson & Johnson). CHT has received research grants (paid to the institution) from ViiV Healthcare, and Gilead Sciences, and consultancy fees and lecture sponsorships from and have served on advisory boards for various laboratories (MSD, Gilead Sciences, ViiV Healthcare, Johnson & Johnson), JR has received research grants (paid to the institution) from Gilead Sciences as well as honoraria for consulting, speaking at educational events and serving on advisory boards from Abbvie, Gilead Sciences, ViiV Healthcare, MSD and Johnson & Johnson. All other authors have no conflicts of interest to disclose.

## Supplementary data

Figures S1A to S1D are available as Supplementary data at *JAC* Online.
