# Supplementary material for: Exploring the pharmacokinetic mechanisms that affect bictegravir exposure during pregnancy
Source: J Antimicrob Chemother. 2026 May 6;81(6):dkag159. doi: 10.1093/jac/dkag159 (PMC13148156; doi:10.1093/jac/dkag159)
Supplement: dkag159_Supplementary_Data [file dkag159_supplementary_data.docx]

## Supplementary material


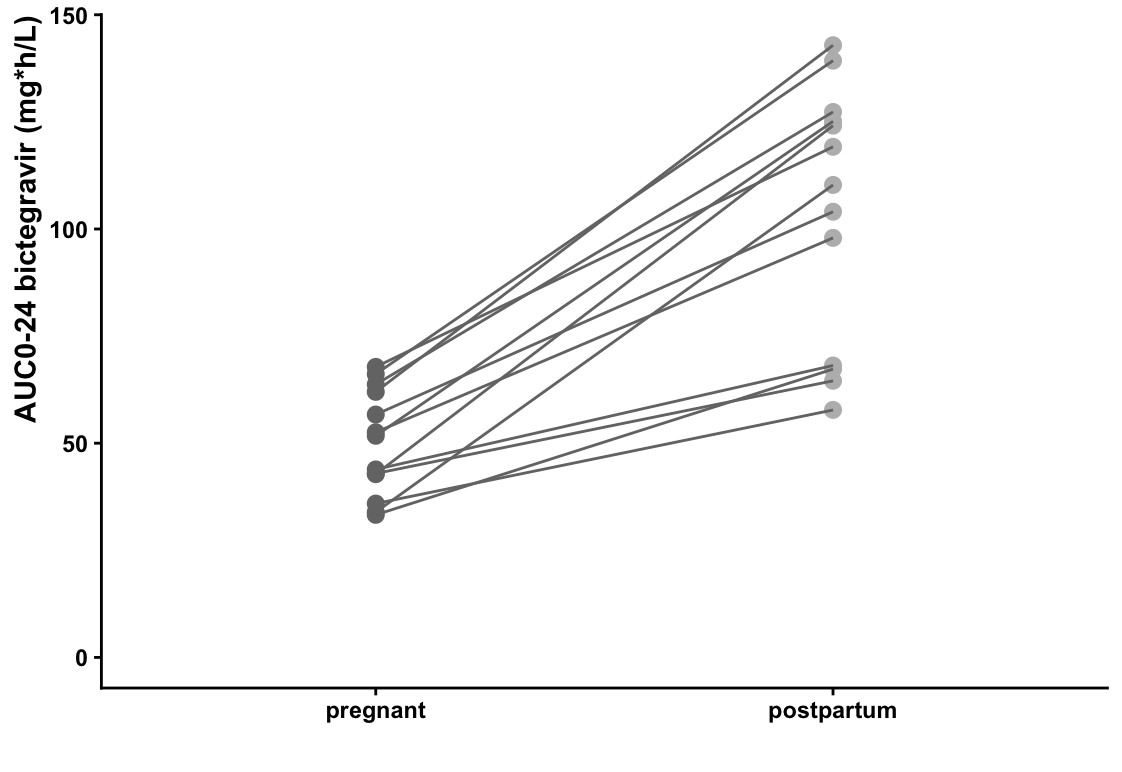


Figure S1A AUC_0-24_ (mg*h/L)of bictegravir in pregnancy and postpartum for each participant


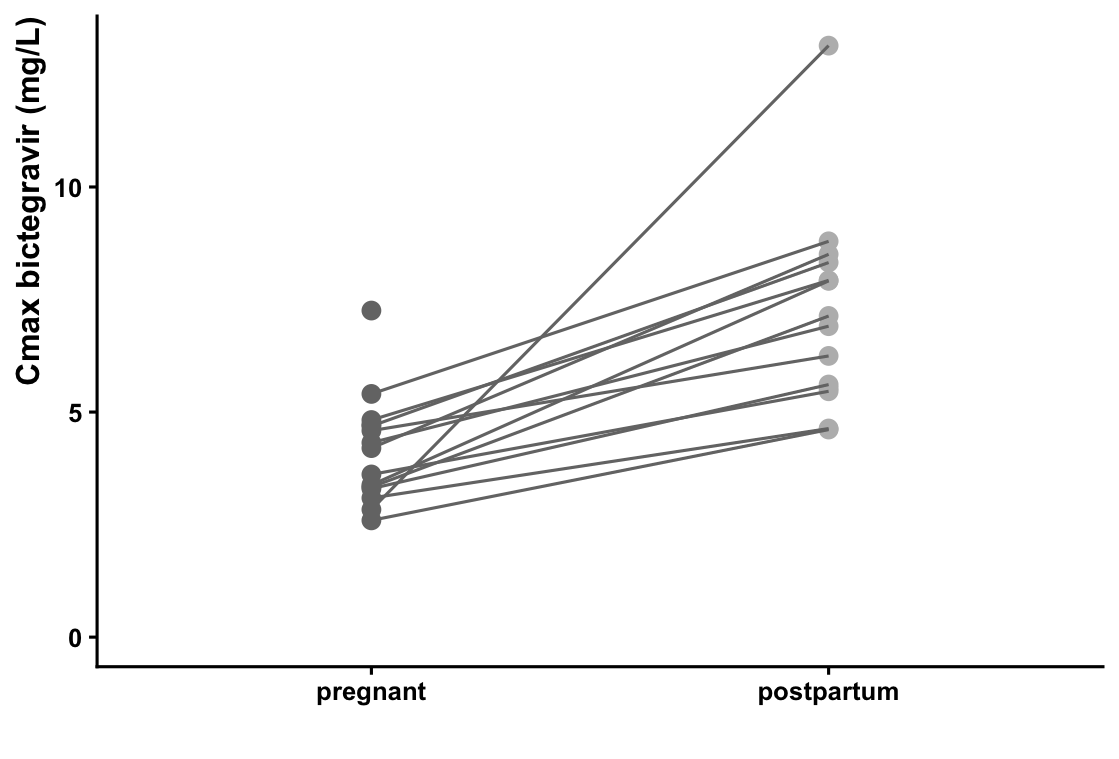


Figure S1B C_max_ (mg/L) of bictegravir in pregnancy and postpartum for each participant


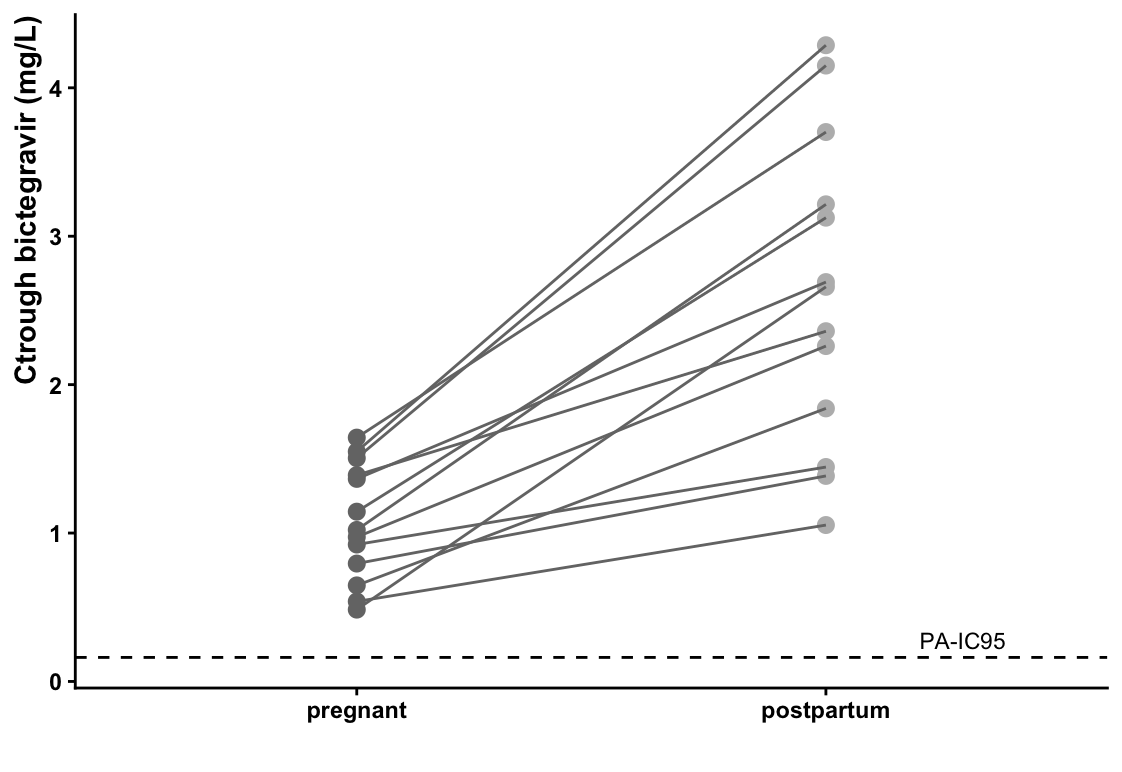


Figure S1C C_trough_ (mg/L) of bictegravir in pregnancy and postpartum for each participant; dashed line represents protein-adjusted IC95 (PA-IC95)


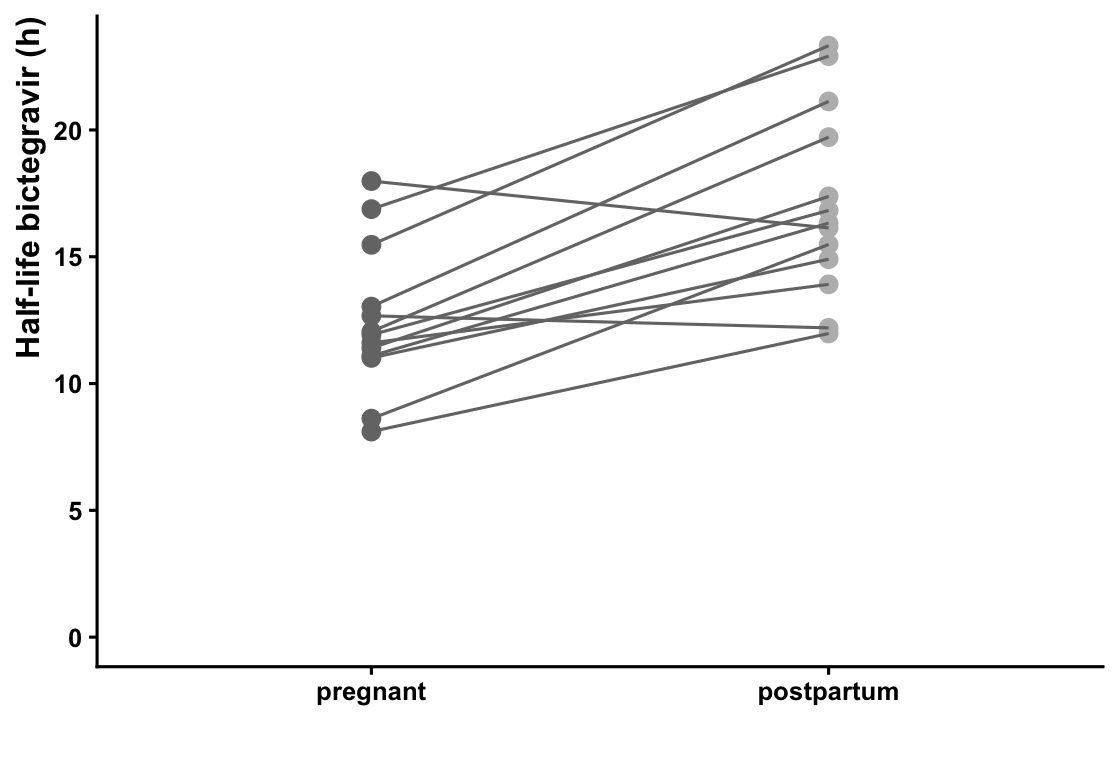


Figure S1D Half-life (h) of bictegravir in pregnancy and postpartum for each participant
